# Communication and information needs about complementary and alternative medicine: a qualitative study of parents of children with cancer

**DOI:** 10.1186/s12906-021-03253-x

**Published:** 2021-03-08

**Authors:** Trine Stub, Sara A. Quandt, Agnete E. Kristoffersen, Miek C. Jong, Thomas A. Arcury

**Affiliations:** 1 Department of Community Medicine, The National Research Center in Complementary and Alternative Medicine (NAFKAM), UiT The Arctic University of Norway, Hansine Hansens veg 19, 9037 Tromsø, Norway; 2 Wake Forest School of Medicine, Department of Epidemiology and Prevention, Division of Public Health Sciences, Medical Center Boulevard, Winston-Salem, NC 27157 USA; 3 Wake Forest School of Medicine, Department of Family and Community Medicine, Medical Center Boulevard, Winston-Salem, NC 27157 USA

**Keywords:** Pediatric cancer, Parents of children with cancer, Complementary medicine, Information and communication needs, Qualitative study: Norway

## Abstract

**Background:**

Many parents choose support such as Complementary and Alternative Medicine (CAM) for themselves and their children who have cancer. The aim of this paper is to describe, how parents who have children with cancer communicated with conventional health care providers about CAM, and what types and sources of information they would like to receive about CAM when the child was ill.

**Method:**

This focused ethnography draws from in-depth, semi-structured interviews conducted with 22 families in Norway with 24 adult participants (two couples), including two individuals who had had cancer themselves. Four domains were explored in the data analysis: the use of CAM, advice from laypeople about CAM, communication with conventional health care providers about CAM, and parents’ information needs about CAM.

**Results:**

Many of the participants had personal experiences with CAM before the child received the cancer diagnosis. The health care providers did not raise the question about CAM in the consultations. However, when the parents raised the question, they were mostly met in a positive way. The participants did not receive any information about CAM at the hospital, which they would have appreciated. Instead, they received recommendations about CAM from laypersons, which were mostly rejected, as the advice was not in line with their health values/philosophy.

**Conclusion:**

The reason participants did not disclose CAM use is that physicians did not ask them about it. However, positive communication about conventional treatment facilitated fruitful conversations about CAM. The participants wanted information about CAM from authoritative sources, primary from health care providers at the hospital and the Children’s Cancer Society. They demand information about risks and benefits when using CAM as well as whether CAM can improve the immune system, fight the cancer, and improve the quality of life of the family. An evidence-based decision aid is warranted to enable health care providers and parents of children with cancer to make well-informed decisions about CAM.

**Supplementary Information:**

The online version contains supplementary material available at 10.1186/s12906-021-03253-x.

## Background

Cancer in children is naturally always an unexpected and demanding diagnosis for parents [1], and it is difficult for many to find reliable information about Complementary and Alternative Medicine (CAM). The user-friendly platforms developed to inform patients, for example CAM Cancer [2] and the website of the United States’s National Cancer Institute, are directed towards adult cancer [3].

Few parents discuss CAM use with the pediatric oncologist [4–6]. The reasons for non-disclosure might be that the parents do not think it is important, or they fear a negative reaction from the oncologist [7]. From a risk perspective and due to possible negative interactions with conventional cancer treatment, it is important to disclose the use of CAM to health care professionals [8]. However, many physicians do not receive training or have little or no knowledge about CAM. They are therefore unable to discuss pros and cons about these modalities with the parents [9, 10]. According to the American Academy of Pediatrics [11], clinicians should avoid dismissal of CAM in ways that communicate a lack of sensitivity or concern for the family’s perspective.

In Norway, the incidence of cancer in children under the age of 15, is about 15 per 100,000 children [12], which is similar to the rest of Europe [13]. More than 300,000 children worldwide are diagnosed with cancer every year [14]. The most common cancers in children are neoplasms of the blood and lymphatic systems, embryonal tumors, and tumors of the brain, bones, and connective tissues [15].

According to parents, symptoms such as pain, emotional distress, fatigue, and loss of appetite cause the most problems for children undergoing cancer treatment [16]. Therefore, many parents choose support such as Complementary and Alternative Medicine (CAM), to reduce cancer treatment-related symptoms in their children [17]. CAM modalities most commonly used are herbs, diet and nutrition, homeopathy, and prayer [4, 18, 19]. CAM is defined as a group of diverse medical and health care symptoms, practices and products that are not generally considered part of conventional medicine [20]. If CAM is used together with conventional medicine, it is considered *complementary,* and if used in place of conventional medicine, it is considered *alternative* [20]. The prevalence of CAM use among children with cancer is high and varies between 6 and 100%, depending on the survey sample and country. The prevalence is on average 47% in high-income countries [21].

Many parents want high quality and reliable information on CAM from authoritative sources [9, 22], preferably provided at the hospital where the children are treated. They also want open, non-judgmental conversations about CAM with the attending physician or oncologist who respects their choices [23, 24]. However, this area of pediatric cancer care has been subjected to limited investigation in Norway. Therefore, we wanted to investigate the use of CAM among parents and children who have had cancer and delineate which communication and information needs they have about CAM.

## Aim

This study draws on qualitative data obtained through in-depth, semi-structured interviews conducted with parents in Norway of children who have been treated for cancer and young adults who themselves were treated for childhood cancer. The aim of this paper is twofold: (1) identify how the participants communicated with conventional health care providers about CAM, and (2) identify types and sources of information they would like to receive about CAM.

## Methods

### Anonymity

In this study, we used pseudonyms and randomized identifier numbers to protect and ensure the anonymities of the participants. According to Norwegian privacy regulations, we are allowed to record age and sex at a group level (the Norwegian Centre for Research Data/ 7,478,928).

### Study area and setting

**Fig. 1:**
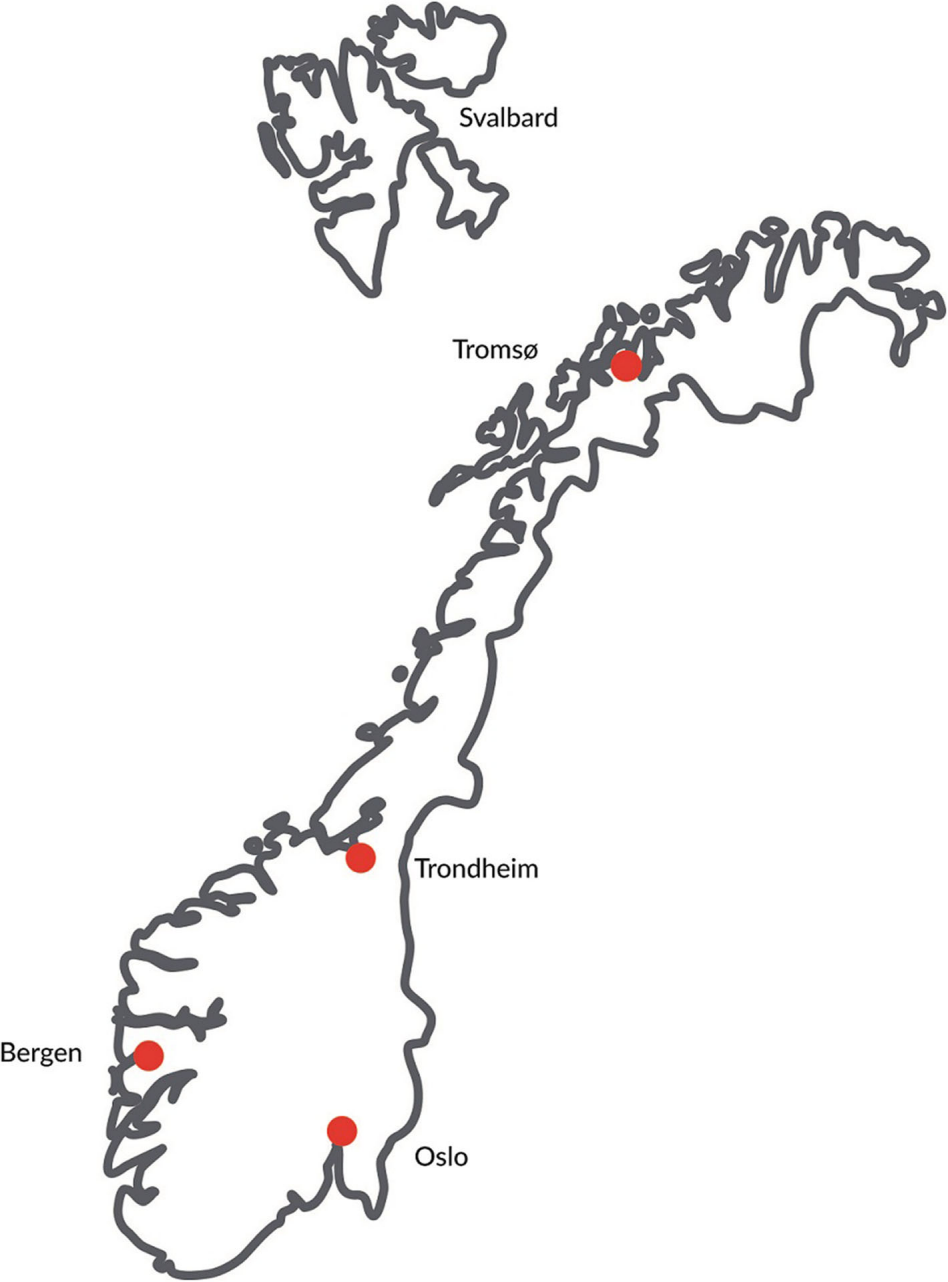
Map of Norway including the four main hospitals for cancer treatment (Oslo, Bergen, Trondheim, and Tromsø, copyright NAFKAM)

Many parents who have children with cancer find it difficult to navigate in a health market where the offer of healing and help is contradictory and complex. The overarching aim of this research project was therefore to generate knowledge and information about CAM that can be helpful for them. This study was conducted as a component of a focused ethnographic study [25] where the investigators wanted to identify parents’ needs regarding the use of CAM. The study took place in Norway. Norwegians receive conventional medical treatment free of charge within the public health care system. According to the Norwegian Health Personnel Act [26], there are 29 occupational groups that are authorized health care professionals in Norway and four main centers/hospitals for treatment of children with cancer. These hospitals are Oslo University Hospital, Haukeland University Hospital in Bergen, St. Olav University Hospital in Trondheim, and University Hospital of North Norway in Tromsø (Fig. 1: Map of Norway).

### Recruitment

Twenty-two families with 24 adult participants (two couples) were recruited to this study. The participants were recruited through Barnekreftforeningen (Children’s Cancer Society). The society [1] is a voluntary, nationwide organization run by parents who have or have had children with cancer. The aims are to be a partner of support and provide reliable information to families who are affected by childhood cancer so that they never feel alone. The society contributes to research and education to combat childhood cancer, and promotes the awareness of childhood cancer in the media. The society has 14 regional offices, all organized and run by parents. These offices also organize social events for families.

Those included were parents/families with a child who have (had) cancer; with any cancer; at any stage; any time since cancer diagnosis. Parents with inadequate Norwegian language skills, or parents who did not understand the meaning or consequences of participating, and parents who were unable to complete the informed consent form were excluded from the study.

**Fig. 2:**
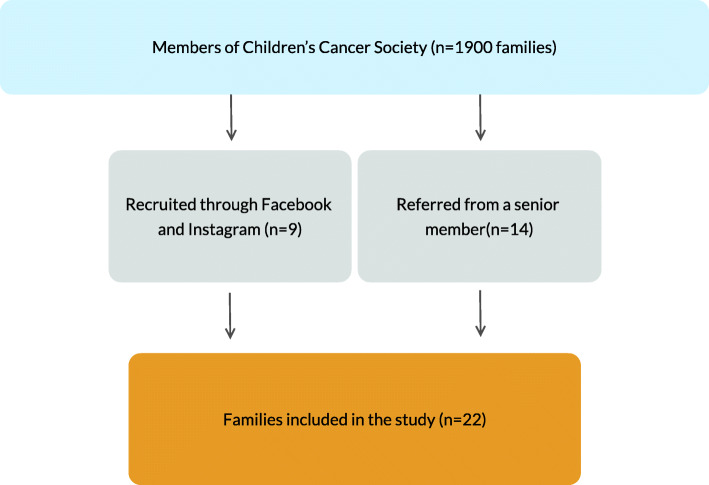
Flow chart of the inclusion process in this study

To recruit participants to the present study, the first author posted invitations on Facebook and Instagram and asked parents to participate in the study. This strategy resulted in nine (*n* = 9) participants. One of the participants gave the researcher contact information to a woman who had been a member of The Children’s Cancer Society for many years. She referred fourteen (*n* = 14) participants to the researcher. Before completing the interviews, the researcher informed the participants about the aim of the study, the purpose, the content of the interviews and her contact information. Written informed consent was obtained from each individual before conducting and recording the interviews. The study participants were informed that they could withdraw from the study for any reason and at any time. A sample of 22 families was perceived enough to achieve saturation (see Fig. 2: Flow chart of the inclusion process in this study).

### Participants

The majority of the participants were females who lived together with a partner. Most of the parents worked in the public sector or health service sector. The cancer-affected children were mostly infants or toddlers when they received a cancer diagnosis (Table 1: Demographic data of the participants). This study was a health service study, so health information about the cancer affected children of the participants was not collected.

**Table 1:** Demographic data of the participants

Parents’ sex	
Female:	(*n* = 20)
Male:	(*n* = 4)
**Child’s sex**	
Female:	(*n* = 13)
Male:	(*n* = 9)
**Age of parents**	
41 years (mean)	18–59 years (range)
**Age of child at diagnosis**	
Infant/toddlers (0–2 years):	(*n* = 10)
Young children (3–9 years):	(*n* = 8)
Teenager (10–19 years):	(*n* = 4)
**Household**	
Married/living as married:	(*n* = 20)
Single parent:	(*n* = 2)
**Occupation**^**a**^	
Health service sector:	(*n* = 8)
Public sector/teacher:	(*n* = 10)
Private sector:	(*n* = 7)
Self-employed:	(*n* = 3)
Disability pension:	(*n* = 2)
**Place of cancer treatment**	
Oslo:	(*n* = 17)
Trondheim, Bergen, or Tromsø:	(*n* = 5)

^a^More options possible

### Data collection

Interviews were semi-structured, and the researcher applied an interview guide developed by the investigators based on an integrated review of the existing literature on parents of children with cancer (see supplementary material). The interview guide was not tested before use. The first author conducted the interviews during the period 1 January 2019 to 31 July 2019. Most of the interviews took an hour to complete. They ranged in length from 30 min to 2 h. The interviews were tape-recorded and took place in a car (*n* = 1), a classroom (*n* = 1), the participants’ home (*n* = 14), hotel rooms (*n* = 2), over the telephone (*n* = 3), as well as at a work place (*n* = 1). Nobody else than the participants were present during the interviews and none repeated interviews was performed. The researcher made field notes during and after the interviews. These interviews were very moving and evoked strong emotions in the participants, as well as in the researcher. Having a child with cancer was a traumatic event for many of the participants, and was experienced as a stroke of fate that affected the whole family.

### Data analysis

Each interview was transcribed verbatim and translated into English as soon as possible following collection. Transcription and translation were completed by professional services. The first author read all transcripts multiple times. She wrote case summaries [27] of topics based on questions from the interview guide and what the parents had expressed in the interviews and synthesized information from different parts of the transcripts. These were reviewed and discussed by the whole team. Information was then sorted across case studies to create a variable-based analysis where the variables were organized into components of three main domains (*advice from laypeople about CAM*, *communication with conventional health care providers about CAM,* and *parents’ information needs about CAM)*. These domains and variables were identified in advanced and during the analysis (see Table 2 for clarification). The team read these, and the analyst returned to the text in an iterative fashion to add more details and to search for commonalities and contrasts across the participants. In particular, the analyst returned to the transcripts to delve deeper into the text to produce descriptions and illustrative quotations about advices from laypeople, communication and information needs about CAM. The transcripts were not returned to the participants for comments.

**Table 2:** Domains and variables identified in the study

Domains identified	Domains	Variables	Variables identified
Before the analytic process	Domain I Advice from laypeople about CAM	Parents contacted by: • CAM provider • Media • Friends/family	Before the analytic process started
Before the analytic process	Domain II Communication with conventional health care providers about CAM	• Needs that were met • Unmet needs	During the analytic process
Before the analytic process	Domain III Parents information about CAM	• Preferred information sources • Preferred type of information	During the analytic process

## Results

This section is organized according to the three main domains (*advice from laypeople about CAM, communication with conventional health care providers about CAM,* and *parents’ information needs about CAM*) derived from the data analysis. Minor domains (*communication needs that were met, unmet communication needs*) will be presented in the communication section and *preferred information sources and types of information* will be presented in the information section.

### Description of the sample

The sample consisted of 22 families with 24 adult participants (two couples), including two adults who were young adults (18 years of age) when they were diagnosed with cancer. The participants’ mean age was 41 years (range from 18 to 59 years). The majority of the participants were women (83%) who held a variety of occupations like teachers, welfare nurses, and self-employees. The households consisted mainly of two parents with two children. Ten children were under the age of 2 years, eight were under the age of 10 and four were in their puberty/teens when they were diagnosed with cancer. The majority of the children (*n* = 17) received treatment at Oslo University Hospital. The other children (*n* = 5) received treatment at the other three centers in Trondheim, Bergen and Tromsø.

Norwegians receive conventional care within the public health system free of charge, while CAM is practiced outside this system, and the patients themselves cover the cost for CAM services. Many (*n* = 11, 46%) of the participants in this study had visited a CAM provider before the children got sick. Others (*n* = 13, 54%) did not consult CAM providers, mainly because they were very satisfied with the treatment they received at the hospital, and the family had agreed to adhere to conventional medicine. Tom (ID11) was generally skeptical of CAM: *There are too many well-intentioned fools and too much profit involved.* Nevertheless, he claimed that CAM definitely has its place in the cancer treatment of children. *The treatment itself and the oncologists are very technically oriented. They [the oncologists] are not the best at psychosocial issues, nor good at talking to relatives. Therefore, it is valuable to have someone else to talk to,* he claimed. Anette (ID14) was somewhat ambivalent about whether CAM should have a place in cancer care. She thought CAM needed to be used in cooperation with the hospital, because she said, *I have* to *be able to trust people that I need to deal with.*

### Domain 1: advice from laypeople regarding CAM

Many parents with children who have had cancer find it difficult to navigate in a health market where the offer of healing and help is contradictory and complex [28]. Seven families received advice from friends and families. Three families were contacted by CAM providers who offered them their services. Recommendations from lay people were often perceived as too much to handle, as they were not in line with the parents’ own health values and philosophy. Consequently, most quotes represent negative experiences, while one positive example is described at the end of this section.

The majority of the participants received advice from lay people (*n* = 13) (ID2, ID3, ID7, ID8, ID9, ID10, ID11, ID12, ID13, ID14, ID15, ID17, ID18). Marianne (ID2) had a friend who told her to give cannabis oil to her child to cure the cancer. The suggestion frustrated her. *I did not discuss it with her [the friend]. I just tried to live my life as normal as possible and have confidence in the recommendations from the oncologists,* she said*.* This family also received a hint from a colleague about a Russian who had made an aerial of aluminum. The idea was to stand between the aerial and a candlelight. The energy from the light was supposed to go through the person and make the person healthy again. The family thought this was stupid and would not expose their child to anything more than the treatment at the hospital. Trude (ID3) was offered healing and tarot card consultations, but she declined. *I just said that we follow our own religion (Roman Catholicism), and then it was ok.* A cousin of her husband told Anette (ID4) that the reason why her child was diagnosed with cancer was that the child had negative thoughts. The cousin told the parents that he and his wife could help them, as they were both healers. Anette still does not speak to that man, as she was very upset about this accusation. Jane’s (ID12) child had been active in the media sharing her cancer history. Therefore, the family received unsolicited advice from several sources. Three healers, among others, offered the child healing. One of them told the mother that he could heal the child, but they had to stop the conventional treatment as he regarded it as poison that would block the body’s ability to heal itself. After that conversation, Jane deleted the healer’s contact information from all her electronic devices. Bente (ID8), on the other hand, experienced that people they knew, family, and friends gave them good advice on CAM, which they appreciated. However, when their advice turned into nagging, she had to put her foot down.

### Domain II: communication with conventional health care providers about CAM

Generally, parents emphasized the importance of an open communication about CAM, as this might avert feelings of frustration and powerlessness that impelled parents to these modalities. Eleven participants (46%) used CAM. Six of them (*n* = 6, 25%) disclosed their CAM use to the health care providers at the hospital (ID3, ID8, ID12, ID14, ID17, ID18), and five participants (*n* = 5, 21%) did not (ID2, ID5, ID6, ID9, ID10). The reasons for withholding disclosure were that the oncologists did not ask, or did not raise the question (ID2, ID5). Marianne (ID2) would have liked the doctor to initiate a conversation about CAM. *This would make it easier to talk about CAM*, she told the interviewer. She would never have initiated such a conversation herself, as she would have felt embarrassed questioning the doctor’s professionalism. Tove (ID9) would have discussed CAM with her doctors if she had felt convinced it would work, and if she could have relied on the doctors to take these questions seriously.

#### Communication needs that were met

The majority of the participants were satisfied with the communication about conventional treatment between them and the health care providers at the hospital. Solveig (ID6) experienced that the communication between herself, her child, and the nurses was good and attentive. Ella (ID20) thought it was frustrating having to deal with so many different doctors, *but maybe that’s the way it has to be*, she said. She claimed, however, that the nurses were like angels. Good communication about conventional treatment facilitated positive conversations about CAM. Bente (ID8) told the oncologist about the use of CAM for her child. She showed the doctor the remedies, and he started laughing and told her they could try. He did not regard these remedies as useful. The mother was happy that the doctor did not turn unfriendly, and, if he had informed her that the combination of these remedies with conventional cancer-treatment could be dangerous, she would have stopped her child from using these. Jane (ID12) discussed the option of a specific diet with the doctors at the hospital, which was approved by the oncologists. Knut and Mari (ID17) told the oncologists and nurses at the hospital that people prayed for them. One of the oncologists told them [the parents] that they tried to meet people, regardless of faith. The parents felt that they were met in a positive way regarding their faith, which made them feel safe. They told: *We are all different. Therefore, we accept that CAM might have a place in cancer care of children, because we need treatments that take care of the whole person. This concept [treat the whole person] differs from person to person,* they said. *Therefore, the health care providers have to meet the needs of every single person.*

Anette (ID14) thought it would be easier being a patient if the walls between the various professions had not been so solid. She would want patient-centered teams, consisting of a physician, nurse, psychologist, and CAM provider. She trusted conventional medicine regarding the treatment of cancer, and she would welcome unambiguous doctors. She experienced that the doctors *only wanted the best for her child*. Nora (ID22) was pleased with the follow-up at the hospital. She would welcome cooperation between the oncologists and homeopaths. She believed that working together would result in proper individual treatments.

#### Unmet communication needs

Grete (ID5) described how she was perceived as a troublesome mother by the hospital staff. During cancer treatment of her child, her job was to provide the best possible care. Mette (ID19) had also mixed experiences of communication with the health care providers. Some oncologists were arrogant and did not want input from the family, whereas others were receptive and attentive. She and her husband wanted to be in control and paid close attention to what happened to their child during treatment. She did not care if some of them [health care providers] did not like her. After a while, there were only nurses in their room, who communicated well with the family. She concluded*: I would do anything for my child, and that is that.* Solveig (ID6) and her husband had their hands full following up the treatment protocol as their child got a lot of medication. When the mother mentioned her concerns about the amount of medication, the oncologist got irritated and told her *that they just had to follow the program*.

Negative communication and experiences at the hospitals about conventional treatment, might be transmitted to other health care areas such as CAM. Silje (ID15) and her husband remembered that a nurse at the hospital advised them to go to Denmark to have *crystal therapy*. According to the nurse, the crystals would light on their child’s body and heal the cells. Silje found this piece of advice incredible, and her husband said: *Well, that [proposal] is probably inappropriate*. Anne (ID1) found Norwegian doctors conservative with the attitude that you do not experiment with children. Therefore, *they are not open to new ways of doing things unless sufficient testing has been performed, and I question how progress can be made,* she explained to the interviewer*.* Moreover, *one of the doctors at the hospital advised us [the family] to follow the protocol and not listen to well-intentioned advice,* Ronja (ID10) explained.

### Domain III: parents information needs about CAM

Autonomy of the family is important when making treatment decisions for children, and adequate, understandable information may empower parents and give them freedom to act on that information. None of the participants in this study received information about CAM at the hospital when their children were sick.

#### Preferred information sources

The majority of the participants wanted information about CAM on the web page of the Children’s Cancer Society (*n* = 8) (ID13, ID11, ID10, ID8, ID7, ID6, ID4, ID20), and/or from health care providers at the hospital (*n* = 13) (ID1, ID2,ID3, ID4,ID5,ID6,ID7, ID9,ID11, ID13, ID14, ID20, ID22). Anne (ID10) would want information about CAM from *health care providers at the hospital*. She had the opinion that the doctors and nurses had little knowledge of CAM, and they got no information about this at the hospital, which she would have appreciated. Marianne (ID2) would want objective evidence-based information from oncologists or other impartial persons (social workers) at the hospital. She would welcome a website about CAM and cancer in children. Trude (ID3) thought that information about CAM could be made available in a folder that can be handed out at the hospital. Mette (ID19) needed no information about CAM when her child was sick. She continued: *At the hospital, we were told not to Google, not to choose CAM, and that is what counts.* However, if she had needed such information she would like the information to be available at the hospital. She would welcome CAM treatment there, such as treatment for nausea, pains, and adverse effects. Tom (ID11) and Trude (ID3) wished for controlled, scientific information about CAM preferably from the *Children’s Cancer Society* (for example a link) or from health care providers. Tom would prefer quality information, which needed to be objective, and based on research and experience. Bente (ID8) said that the Children’s Cancer Society could have a link to a page containing objective information. Ingrid (ID16) was not negative to CAM. Nevertheless, she was skeptical. Sondre, her husband, failed to see the need for offering information about CAM in general.

#### Type of information

Marianne (ID2) and Anne (ID1) would want information on whether CAM might cure cancer, improve the immune system, information about the effect of CAM on the cancer itself (in the absence of conventional treatment) and whether it can increase the chances of survival.

Anne (ID1) thought the information about CAM should be objective and focus on reducing adverse effects and information about possible negative interactions with conventional treatment. Nora (ID22) would welcome further research on CAM and cancer and a possible modality with less late effects compared to today’s conventional treatment. Children have a developing body, and cancer treatment have more or less strong adverse effects. Several of the participants were therefore concerned about late effects of the cancer treatment and would appreciate further research on late effects (ID4, ID10, ID22). Jane would like information of long-term effects of cancer treatment such as harvesting of ovarian eggs, and why influenza vaccines are important.

Several of the participants wanted information about how to increase quality of life*.* Ella (ID20) demanded information about things that can make daily life easier for the children. Marianne (ID2) wanted information about how to increase quality of life, including how to support the family, how to support children who are afraid, and what to do for yourself when you get anxious. She claimed that parents need support to find out about all of this.

Jane (ID12) explained: *When your child gets cancer, you don*’*t know what to ask.* Therefore, she would want a folder containing information about CAM in general, and more specifically about D-vitamins. Ella (ID20) would also welcome information about which vitamins and minerals that can be used. Many doctors advised the parents not to give antioxidants to their children to strengthen their immune system. The rationale was that when suffering from cancer, herbs or supplements that increase the count of white blood cells should not be given, as this count is already too high. Many parents found this information important, and they thought that this information should be spread to others parents. Sofie (ID4) would want information about reflexology and nutritional needs.

Mette (ID19) thought that great caution should be exercised when giving CAM to children with cancer. *It must be safe to use, mostly to provide relief rather than an additional burden, and approved by conventional doctors,* she said. Essentially, she was open to CAM, but when it came to her own child, she needed to be absolutely sure that it would not hurt, and that it would help.

## Discussion

Many of the participants (*n* = 11, 46%) in this study used CAM for themselves, and when they disclosed their children’s use of CAM, the oncologists were mostly positive. However, some were very skeptical and recommended against such use. The reason for withholding disclosure was that the oncologist did not ask.

The participants did not receive any information about CAM at the hospital, which they would have appreciated. Instead, they were advised not to use CAM and not to take into consideration well intended advice from family and friends. The majority of the participants in this study received advice about CAM from lay people. Many of these recommendations were rejected, as they were not in line with the parents’ health values.

The majority of the participants (*n* = 21, 95%) wanted evidence-based information about CAM from authoritative sources such as health care providers at the hospital or from the Children’s Cancer Society. They wanted information about vitamins and supplements, how to reduce adverse effects of chemotherapy, improve the immune system, and fight the cancer. Moreover, they wanted information about how to support the families, how to cope with life, and reduce anxiety for themselves and for their children.

### Use of CAM

The National Research Center in Complementary and Alternative Medicine (NAFKAM) study from 2016 [29] found that 5% in an unselected Norwegian population visited a CAM provider for their children’s health problems during a year. About 15.7% of the parents gave their children supplements, herbs, or natural health products. Only a handful of the children used self-help techniques. A Norwegian study from 2007 [30], reported that parents were very restrictive about giving supplements and natural remedies to their children with cancer, but more positive regarding their own use.

A study from The Oslo University Hospital showed that 53% of the patients used CAM products to strengthen the immune system of the child. Only 18% thought that such products would contribute to the inhibition or cure of the cancer, whereas 62% did not know [28]. Another Norwegian study [31] demonstrated that CAM treatment was provided either simultaneously with the hospital treatment or when the patients were referred to palliative care. Moreover, the parents’ motivation for CAM use seemed to be focused on the basic need of actively taking part in saving their children’s lives, knowing they had tried everything [31].

### Advice about CAM

The majority of the participants (*n* = 13, 54%) in our study received well-intended advice about CAM from family and friends, which was mostly perceived as a burden and therefore rejected. However, according to Gilmour [32], the reason for using family and friends as the main source of information was that they did not receive CAM information from the oncologists. Fernandez et al. [33] also reported that parents received information on CAM from families and friends*.* This is in accordance with Gozum et al. [34] who reported that most parents in Turkey learn about CAM from friends and relatives or other families with children who have cancer.

### Studies about communication of CAM

Parents and patients with cancer highly value the input from physicians about CAM [30, 35, 36]. Ideally, they should feel free to discuss all options without the fear of being rejected. This can best be achieved through open, transparent, non-judgmental, and informed discussions about possible outcomes of combining CAM and conventional treatment for cancer [37, 38]. Between 38 and 60% of cancer patients, however, use CAM without informing their healthcare teams [39]. The reason for withholding disclosure was that the oncologists did not ask. The parents in this study reported that positive communication about conventional treatment facilitated fruitful conversations about CAM. If they talked with the treating oncologists about CAM were met in a positive way. Compared to previous research in Norway [37], this demonstrates a positive change in health providers’ attitude towards CAM.

Health care providers provide ethical care by respecting parents’ choice on using CAM for their child [40]. The importance of keeping hope alive when the child is serious ill is important [7, 40, 41]. In order to try all possible modalities for their child and maintain hope, parents often perceive CAM safer and more efficient than research demonstrates [7, 40]. Gagnon and Recklitis [42] investigated how the parents’ preferred level of control in treatment decision making was related to their personal health care involvement and their decision to use CAM for their children. They found that most parents using CAM preferred active or collaborative versus passive decision-making.

### Studies about information on CAM

There is a need among parents of children with cancer for information about CAM. Studies demonstrates that the most common reason for not using CAM is lack of information [33, 43] and additional stress for the child [44]. This is in line with the findings from our study where the participants had limited capacity to search for information regarding CAM because following up the conventional treatment protocols was time consuming. This is in line with Fletcher et al. [24] who found in a qualitative interview study that parents wanted information about CAM integrated in the services they already received in the hospital, as lack of time hindered them from examining CAM modalities themselves. In a survey Ben-Arush et al. [45] reported that the majority of parents were very interesting in obtaining more information and guidance about these modalities. Krogstad et al. [30] found that the participants had not received any information from the oncologists or other health care providers at the hospital about CAM products, which they were very interested in obtaining. Moreover, information about CAM may give the parents a sense of control of the child’s treatment. It may also provide additional ways of helping their child to get through his/her cancer treatment. Finally, it may give parents the feeling that they are doing everything possible to support their child’s recovery [43].

### Strengths and limitations

Qualitative analysis provides insights into how participants understand and interpret situations, but it cannot be used to establish associations [25, 46]. This study should therefore be interpreted in light of its strengths and limitations. Twenty-two families agreed to be part of an interview. Having more than 22 interviews may have resulted in a richer variety in experiences. However, no further substantial variation was added during the final three interviews, leading to the judgment that the information power was sufficient and that a larger number of interviews would not have significantly altered the outcome of the thematic analysis [47]. Moreover, the interviews were of much depth, and the material was therefore very rich, and the participants demonstrated striking similarities in their personal history, concerns and strategies. Another strength of this study was that the interviewed parents were geographically from all over Norway. However, the study is based on data from a selected group of parents, as all were recruited via The Children’s Cancer Society. Therefore, the present findings may not be representative for all parents of children with cancer in Norway.

## Conclusion

The reason participants did not disclose CAM use is that physicians did not ask them about it. However, positive communication about conventional treatment facilitated fruitful conversations about CAM. Parents of children with cancer need information about CAM from authoritative sources. An evidence-based decision aid is warranted to enable health care professionals and parents of children with cancer to make well-informed decisions about CAM.

## Supplementary Information

**Additional file 1.** Interview guide, families with children/adolescents who have had cancer.

## Data Availability

The raw dataset is not available publically due to Norwegian privacy regulations. Applicants for any data must be prepared to conform to Norwegian privacy regulations. Researchers, who want to request the data, can contact the first author.

## Abbreviation

CAM: Complementary and Alternative Medicine
