# Supplementary material for: Communication and information needs about complementary and alternative medicine: a qualitative study of parents of children with cancer
Source: BMC Complement Med Ther. 2021 Mar 8;21:85. doi: 10.1186/s12906-021-03253-x (PMC7938468; doi:10.1186/s12906-021-03253-x)
Supplement: Supplementary file 1 — Additional file 1. Interview guide, families with children/adolescents who have had cancer. [file 12906_2021_3253_MOESM1_ESM.docx]

**Interview guide, families with children/adolescents who have had cancer**

**Introduction**

**Tell about yourself: Age, education, work, housing (house, apartment, farm) and domicile (town, countryside)**

**Your family consists of: Partner, children, pets, activities**

**What is your story about the cancer disease?**

**Key questions**

- **What is your experience of using alternative treatment (AB)? Does your family have a history of using alternative treatment to cure illness?**
- **Personal/Your partner´s use of AB: Prior to/After the child was diagnosed with cancer.**
- **The child´s use of AB: Prior to/After the diagnosis**
- **What kind of alternative treatment did you use when the child was ill?**
- **For how long did the alternative treatment last?**
- **What was the reason for choosing AB when your child was ill? Did your child have certain symptoms that you wanted to cure using alternative treatment?**
- **Who suggested this treatment?**
- **Rationale for use of alternative treatment: Symptom relief, strengthen the immune system, hope, try everything, symptom reduction**

***Research shows that parents often talk about the need to be involved in their children’s treatment experience and described feeling huge responsibility for ensuring their children received appropriate care.***

- **Rationale for not using alternative treatment (Well satisfied with the doctor´s/hospital´s treatment.)**
- **In your opinion, was the AB treatment effective? If yes, what was the actual effect of the AB treatment?**
- **Did you experience adverse effects from the AB treatment?**
- **In your opinion, what are the disadvantages of using alternative treatment? (Adverse effects, lack of research on effect, other reasons.)**

**Information**

- **Were you contacted by alternative therapists/the media/friends and family about the use of alternative treatment when your child was ill?**
- **Did you feel pressured to use AB for your child by friends/family/the media. Please elaborate**
- **Were you in need of information about alternative treatment and childhood cancer?**
- **Where did you get information about AB?**
- **What made you choose the specific method/therapist that you did?**
- **Please tell about the process leading to your choice.**
- **Did you receive information from the doctor, hospital, alternative therapist?**
- **What kind of information about AB would have been useful for you when your child was ill?**
- From whom and where do you wish to receive information about alternative treatment and childhood cancer?

**-Do parents who consider using AB for their children with cancer sufficient and correct information?**

**What is possibly lacking?**

**-Who should be responsible for producing and providing parents with such information? What should it contain?**

**Where should such information be available so that parents can easily access it?**

**Communication with doctors**

- **Did you address the use of alternative treatment for the child with your doctor/health personnel when your child was ill?**
- **If yes, what was their reaction? Did they discourage or support the use of AB?**
- **If no, what was your reasons for not addressing this issue with the doctor?**
- **What kind of information do you need to be able to discuss alternative treatment with your doctor/health personnel?**
- How would you like to receive this information. Type, form, general, personal.

**Communication with alternative therapists**

- **Did you find it easy to find an alternative therapist for your child?**
- **Please tell about this process?**
- **Did the AB therapist inform you of what he/she could do for you? Information about the treatment.**
- **What kind of advice did you receive about conventional treatment (follow standard, medical procedures, postpone conventional treatment, combine AB and conventional treatment, other?**
- **Were you informed of possible interactions between the AB treatment and conventional treatment?**
- **Adverse effects from AB**
- **Do you know if there was any communication/contact between the AB therapist and the doctor/health personnel about the use of AB while your child was ill?**
